# Cardiac resynchronization therapy with or without atrioventricular node ablation in atrial fibrillation: the CAAN-AF trial

**DOI:** 10.1093/eurheartj/ehag206

**Published:** 2026-04-16

**Authors:** Prashanthan Sanders, Jonathan P Ariyaratnam, Martin K Stiles, Alexis Puvrez, Stephen J Nicholls, Walter P Abhayaratna, Mark J Perrin, Jeff Alison, Rajeev K Pathak, Anand Ganesan, Vincent Paul, Stuart P Thomas, Gijo Thomas, Melissa E Middeldorp, Elnaz Shahmohamadi, Adrian D Elliott, Robert Park, Haris Haqqani, Dean Boddington, Peter M Kistler, Paul Martin, Raymond W Sy, Stephan Willems, Paul Stobie, Matthew Webber, Gerald Kaye, Paul MacIntyre, Bobby John, Daniel Steven, Azlan Hussin, Suresh Singarayar, David Whalley, David O’Donnell, Nigel Lever, Rajiv Mahajan, Daniel Ninio, John Amerena, Adrian Esterman, Derek P Chew, Matthew Worthley, Andrew Tonkin, Carmine G De Pasquale, Jonathan M Kalman

**Affiliations:** Centre for Heart Rhythm Disorders, Adelaide University, Adelaide, South Australia, Australia; Department of Cardiology, Central Adelaide Local Health Network, Adelaide, South Australia, Australia; Centre for Heart Rhythm Disorders, Adelaide University, Adelaide, South Australia, Australia; Department of Cardiology, Central Adelaide Local Health Network, Adelaide, South Australia, Australia; Waikato Clinical School, University of Auckland, Hamilton, New Zealand; Centre for Heart Rhythm Disorders, Adelaide University, Adelaide, South Australia, Australia; Victorian Heart Institute, Monash University, Melbourne, Victoria, Australia; Department of Medicine, Canberra Hospital and the Australian National University, Canberra, Australian Capital Territory, Australia; Department of Cardiology, University Hospital, Geelong, Victoria, Australia; Waikato Clinical School, University of Auckland, Hamilton, New Zealand; Canberra Heart Rhythm, Canberra, Australian Capital Territory, Australia; Department of Cardiology, Flinders University and Flinders Medical Centre, Adelaide, South Australia, Australia; Department of Cardiology, Fiona Stanley Hospital, Perth, Western Australia, Australia; Department of Cardiology, Westmead Hospital, Sydney, New South Wales, Australia; Centre for Heart Rhythm Disorders, Adelaide University, Adelaide, South Australia, Australia; Centre for Heart Rhythm Disorders, Adelaide University, Adelaide, South Australia, Australia; Department of Cardiology, Central Adelaide Local Health Network, Adelaide, South Australia, Australia; Centre for Heart Rhythm Disorders, Adelaide University, Adelaide, South Australia, Australia; Centre for Heart Rhythm Disorders, Adelaide University, Adelaide, South Australia, Australia; Department of Cardiology, Central Adelaide Local Health Network, Adelaide, South Australia, Australia; Department of Cardiology, Gold Coast Hospital, Gold Coast, Queensland, Australia; Department of Cardiology, Prince Charles Hospital, Brisbane, Queensland, Australia; Department of Cardiology, Tauranga Hospital, Tauranga, New Zealand; Department of Cardiology, Alfred Hospital, Melbourne, Victoria, Australia; Department of Cardiology, Royal Brisbane Hospital, Brisbane, Queensland, Australia; Department of Cardiology, Royal Prince Alfred Hospital & Concord Repatriation General Hospital, Sydney, New South Wales, Australia; Department of Cardiology, Asklepios Clinic St.Georg, Hamburg, Germany; Department of Cardiology, Sir Charles Gairdner Hospital, Perth, Western Australia, Australia; Department of Cardiology, Capital & Coast Wellington Hospital, Wellington, New Zealand; Department of Cardiology, Princess Alexandria Hospital, Brisbane, Queensland, Australia; Department of Cardiology, Royal Hobart Hospital, Hobart, Tasmania, Australia; Department of Cardiology, Townsville Hospital, Townsville, Queensland, Australia; Department of Electrophysiology, University Hospital Cologne, Cologne, Germany; Department of Cardiology, National Heart Institute, Kuala Lumpur, Malaysia; Department of Cardiology, Prince of Wales Hospital, Sydney, New South Wales, Australia; RDepartment of Cardiology, oyal North Shore Hospital, Sydney, New South Wales, Australia; Department of Cardiology, Austin Health, Melbourne, Victoria, Australia; Department of Cardiology, Auckland City Hospital, Auckland, New Zealand; Centre for Heart Rhythm Disorders, Adelaide University, Adelaide, South Australia, Australia; Department of Cardiology, Lyell McEwin Hospital, Adelaide, South Australia, Australia; Department of Cardiology, Central Adelaide Local Health Network, Adelaide, South Australia, Australia; Department of Cardiology, University Hospital, Geelong, Victoria, Australia; College of Health, Adelaide University, Adelaide, South Australia, Australia; Victorian Heart Institute, Monash University, Melbourne, Victoria, Australia; Department of Cardiology, Flinders University and Flinders Medical Centre, Adelaide, South Australia, Australia; Centre for Heart Rhythm Disorders, Adelaide University, Adelaide, South Australia, Australia; Department of Cardiology, Central Adelaide Local Health Network, Adelaide, South Australia, Australia; Department of Epidemiology and Preventative Medicine, Monash University, Melbourne, Victoria, Australia; Department of Cardiology, Flinders University and Flinders Medical Centre, Adelaide, South Australia, Australia; Department of Cardiology, Melbourne Health and University of Melbourne, Melbourne, Victoria, Australia

**Keywords:** Cardiac resynchronization, Heart failure, AV node ablation, Rate control

## Abstract

**Background and Aims:**

Patients with heart failure with reduced ejection fraction (HFrEF) derive less benefit from cardiac resynchronization therapy (CRT) if they have coexisting atrial fibrillation (AF). Observational data suggest that atrioventricular node ablation (AVNA) may enhance CRT efficacy in this population. This trial aimed to evaluate the effect of AVNA compared with medical rate control therapy (MRCT) on CRT outcomes in patients with HFrEF and permanent AF.

**Methods:**

The CAAN-AF trial was an international, prospective, multicentre, randomized controlled trial evaluating the impact of AVNA in patients with HFrEF, permanent AF, and CRT-defibrillators (CRT-D). Participants were randomly assigned (1:1) to AVNA or MRCT targeting a resting heart rate <90 bpm. All participants received device optimization. The primary endpoint was a composite of all-cause mortality and nonfatal heart failure events. Secondary endpoints included cardiovascular mortality, unplanned hospitalizations, ventricular arrhythmias requiring device therapy, 6-min walk distance (6MWD), and quality of life.

**Results:**

With early termination of the trial due to futility, a total of 143 patients were randomized (67 to AVNA, 76 to MRCT). Baseline characteristics were similar across groups. No significant difference was found in the primary endpoint [47 vs. 46 events; incident rate ratio (IRR), 1.16; 95% confidence interval (CI), 0.60–2.24]. Secondary outcomes, including cardiovascular mortality (odds ratio, 1.93; 95% CI 0.60–6.20), unplanned hospitalizations (IRR, 1.01; 95% CI 0.71–1.74), ventricular arrhythmias requiring device therapy (IRR, 0.68; 95% CI 0.17–2.63), 6MWD, and SF-36 scores, also showed no significant differences.

**Conclusions:**

This randomized trial of patients with permanent AF and CRT-D found no evidence of a reduction in all-cause mortality and nonfatal heart failure events with AVNA compared with MRCT.

**Trial Registration:**

NCT01522898.

## Structured Graphical Abstract

**Structured Graphical Abstract:**
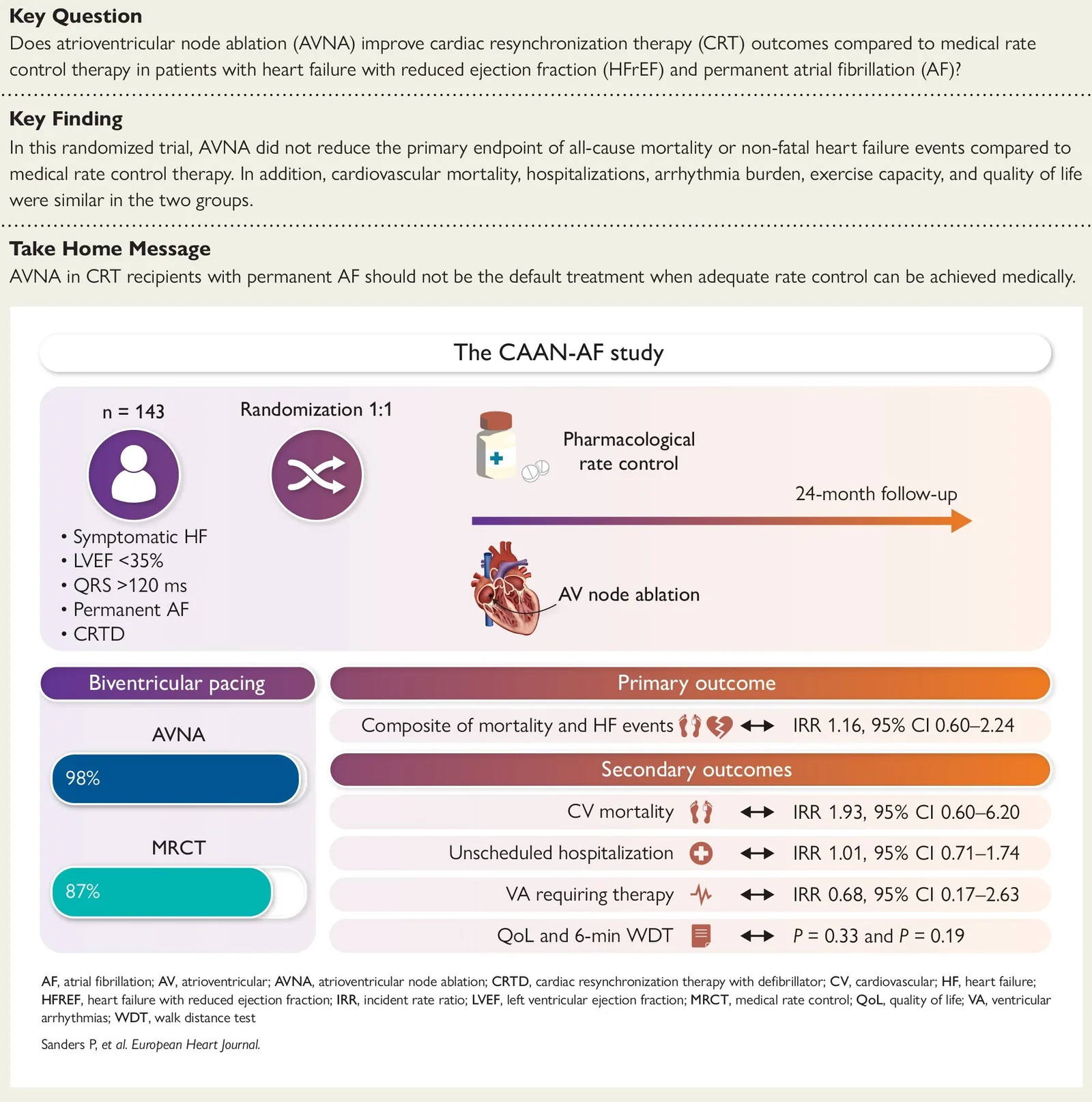

**See the editorial comment for this article ‘Cardiac resynchronization therapy in atrial fibrillation: do all patients need atrioventricular node ablation?’, by M. Brignole, https://doi.org/10.1093/eurheartj/ehag355.**

## Introduction

Cardiac resynchronization therapy (CRT) is a cornerstone of heart failure (HF) management in patients with severely reduced left ventricular ejection fraction, HF symptoms [New York Heart Association (NYHA) class II–IV] and electrical dyssynchrony.^1^ Several randomized clinical trials have demonstrated benefit of CRT, including reductions in mortality and hospitalizations, as well as improvements in symptoms and quality of life.^2–7^ However, the benefits of CRT in patients with atrial fibrillation (AF) are less well-defined. While 40% of CRT-eligible patients have AF,^8^ patients with AF were largely excluded from the clinical trials investigating CRT. Observational data suggest that AF may attenuate the response to CRT, with these patients demonstrating worse outcomes with CRT compared with patients without a history of AF.^9–12^

A key factor proposed for the reduced efficacy of CRT in patients with AF is the lower percentage of effective biventricular pacing in this population, due to preserved intrinsic conduction through the atrioventricular node.^13,14^ As a result, atrioventricular node ablation (AVNA) has emerged as a potential strategy to enhance CRT efficacy in patients with AF and is currently given a Class IIa recommendation for the management of AF patients eligible for CRT.^15^ Observational studies suggest that in patients with a CRT-defibrillator (CRT-D) and AF, AVNA may significantly reduce mortality and improve clinical outcomes.^16^ Despite these promising findings, evidence from randomized controlled trials remains lacking.

In the Cardiac Resynchronization Therapy And Atrio-Ventricular Node Ablation Trial in Atrial Fibrillation (CAAN-AF) randomized clinical trial, we sought to investigate this evidence gap by comparing AVNA with medical rate control therapy (MRCT) in CRT-D eligible patients with permanent AF.

## Methods

### Trial design and oversight

The CAAN-AF trial was a Phase IV international, investigator-initiated, multicentre, prospective, randomized clinical trial in which patients with HF with reduced ejection fraction (HFrEF; in the study defined by a left ventricular ejection fraction ≤35%), QRS duration ≥120 ms, and permanent AF with a CRT-D device were randomized to receive either AVNA or MRCT. The trial was carried out in the hospital setting across 28 centres in four countries. The trial protocol was reviewed and approved by the local Human Research Ethics Committee at each participating centre. The full trial protocol is provided in the Supplementary data online.

The steering committee designed and oversaw the conduct of the trial and the analysis of the data. An independent and blinded Clinical Event Committee reviewed and adjudicated all mortality, morbidity, and adverse event data. The study safety was reviewed by an independent Data Safety Monitoring Board (DSMB). Full details of the trial design have been previously published.^17^ Trial Registration at ClinicalTrials.gov (ID: NCT01522898).

### Participants

Patients were eligible for enrolment if they were at least 18 years of age and had stabilized HF with NYHA Class II and III or ambulatory Class IV symptoms, left ventricular ejection fraction ≤35%, and QRS duration ≥120 ms on maximally tolerated, guideline-directed medical therapy (GDMT). In addition, patients were required to have a diagnosis of permanent AF. Patients were enrolled either before (Pathway A) or after (Pathway B) CRT-D implantation, where AF had developed after implantation of CRT-D (see Supplementary data online, *Figure S1*). All patients met international guideline criteria for the implantation of CRT-D devices. However, patients who improved to NYHA Class II HF symptoms after CRT implant remained eligible for inclusion into this study. Full details of the inclusion and exclusion criteria are provided in Supplementary data online, *Table S1*.

### Enrolment and randomization

Eligible patients agreeing to enrol in the study provided written, informed consent to participate. Randomization was undertaken at least 30 days after CRT-D implantation to allow for optimization of CRT. Randomization was performed using a computer-generated web-based randomization schedule and stratified by trial centre using randomly permuted blocks of sizes 2 and 4. The randomization schedule was incorporated within the online data management software.

### Guideline-directed medical therapy

Optimization of GDMT was supervised by the treating cardiologist at each participating site. GDMT was recommended according to the European Society of Cardiology guidelines at the time of trial registration.^18^ This included use of an angiotensin-converting enzyme inhibitor (ACEi) or angiotensin receptor blocker (ARB), beta-adrenoceptor blocker, and mineralocorticoid receptor antagonist (MRA). The treating physician was responsible for titrating these medications to achieve maximum tolerated doses.

### Cardiac resynchronization therapy defibrillator implantation and programming

Participants in whom there was no pre-existing CRT-D (Pathway A) underwent CRT-D implantation at their local participating trial centre. This pathway included patients undergoing upgrade to CRT-D from single- or dual-chamber pacemakers or implantable cardiac defibrillators (ICD). Details of the CRT-D implantation procedures are presented in Supplementary data online. Participants in Pathway A were randomized 30 days following CRT-D implantation, during which time further optimization of device programming could take place. Lower rate programming was set between 60 and 80 bpm in accordance with the physician’s preference. Participants with a pre-existing CRT-D could be enrolled and randomized immediately provided the device had been implanted and optimized more than 30 days prior (Pathway B). Details regarding CRT-D programming are provided in Supplementary data online, *Methods S1* and Supplementary data online, *Table S2*.

### Atrioventricular node ablation: intervention arm

Atrioventricular node ablation was performed for all participants randomized to the intervention arm. The ablation was undertaken as close to randomization as possible and in all cases was performed within 6 weeks of randomization. Full details of the AVNA procedures are provided in Supplementary data online.

### Medical rate control therapy: control arm

Patients in the control arm received pharmacotherapy for MRCT. Physician preference directed the choice of drugs to achieve MRCT with a preferential order of beta-blockade, then digoxin, then amiodarone if necessary. The use of calcium channel blockers was discouraged given the presence of left ventricular dysfunction. The optimal target for heart rate control was a resting heart rate of <90 bpm.

### Follow-up

Patients were followed up for a period of 24 months after randomization. In-person trial visits were scheduled at 6, 12, 18, and 24 months post-randomization. Assessments undertaken at each of these timepoints are shown in Supplementary data online, *Table S3*. Crossovers between groups were only permissible if the participant had already experienced a nonfatal HF event and thus has reached the primary endpoint, to minimize dilution of power for the primary analysis.

### Trial endpoints

The primary endpoint for this study was the number of deaths and nonfatal HF events, defined as signs/symptoms of HF requiring intravenous diuretics or inotropes, at 24 months of randomization. Secondary endpoints included the individual components of the composite primary endpoints in addition to cardiovascular mortality, unplanned hospitalizations, ventricular arrhythmia requiring device therapy, inappropriate defibrillator shocks, biventricular pacing percentage, 6-min walk distance (6MWD), HF symptoms based on the Minnesota Living with Heart Failure (MLHF) questionnaire, and quality of life according to the SF-36 questionnaire. Further details of the MLHF and SF-36 questionnaires are provided in the Supplementary data online.

### Sample size calculation

The trial was powered to test the potential superiority of AVNA over MRCT. A previous meta-analysis of observational data suggested that AVNA reduces all-cause mortality by 58%.^16^ Another individual patient meta-analysis of five randomized CRT trials reported a hazard ratio of 0.65 [95% confidence interval (CI) 0.58–0.74] for all-cause mortality and nonfatal HF events.^19^ Acknowledging the risk of overestimating the effect size by using nonrandomized data, a conservative stance was taken, and this trial was therefore powered to identify a 25% relative reduction in event rate.

The event rate in the control arm was estimated at 50 events per 100 person-years of follow-up, based on the results of the COMPANION trial of CRT.^2^ To achieve 80% power to detect a 25% reduction in event rate at an alpha level of 5% (two-sided Type 1 error), assuming 1:1 randomization and a 10% crossover rate, it was estimated that 295 participants would be required for each treatment arm, translating to a total of 590 participants.

In light of the compelling observational data supporting AVNA in patients with CRT-D and AF,^16^ interim analyses were prespecified with predefined stopping boundaries for efficacy at 25%, 50%, and 75% of the recruitment target. The formal stopping criteria were determined according to the method of O’Brien and Fleming.^20,21^ The criteria to reject the null hypothesis of no difference between treatment groups at three interim analyses were, respectively, 2-tailed *P*-values < .000015, .0031, and.014, with *P* < .044 for the final analysis of the primary outcome. The DSMB requested an additional conditional-power futility analysis at the first interim analysis after it had identified nearly identical event rates across both groups.

### Statistical analyses

All analyses were conducted under the intention-to-treat principle. The primary outcome of the total number of composite events (all-cause mortality and nonfatal HF) between AVNA and MRCT groups was analysed using negative binomial regression model and reported as incident rate ratio (IRR) with 95% CIs. All-cause and cardiovascular mortality between the AVNA and MRCT groups were analysed using logistic regression and reported as odds ratio (OR) with 95% CIs. Total all-cause unplanned hospitalizations, ventricular arrhythmias, and inappropriate shocks were analysed using negative binomial regression and reported as IRR with 95% CIs. Quality of life data from SF-36 v2 Health Survey and MLHF questionnaire were analysed using mixed effects model for repeated measures with the baseline score as a covariate. For the purposes of these analyses, missing data were assumed to be missing at random. The statistical analysis plan is provided in Supplementary data online.

## Results

### Study population

Between 22 May 2013 and 29 July 2020, 436 patients were screened for inclusion. A total of 293 patients were excluded for prespecified exclusion criteria; accordingly, 143 patients were randomized at 28 centres from four different countries (see Supplementary data online, *Table S4*).

The first interim analysis was performed on the basis that 24.2% of the target sample size had been recruited with a median follow-up of 24 months.^23,24^ This analysis identified nearly identical event rates across both groups, and, therefore, a formal futility analysis was performed. This futility analysis yielded a conditional power for successful completion of the study of 2.7%. Noting that the conditional power was <20% and that continued recruitment could not be justified, the DSMB recommended early termination of the trial to which the Steering Committee unanimously agreed. Further details of the futility analysis are provided in Supplementary data online, *Table S5*.

**Figure 1:**
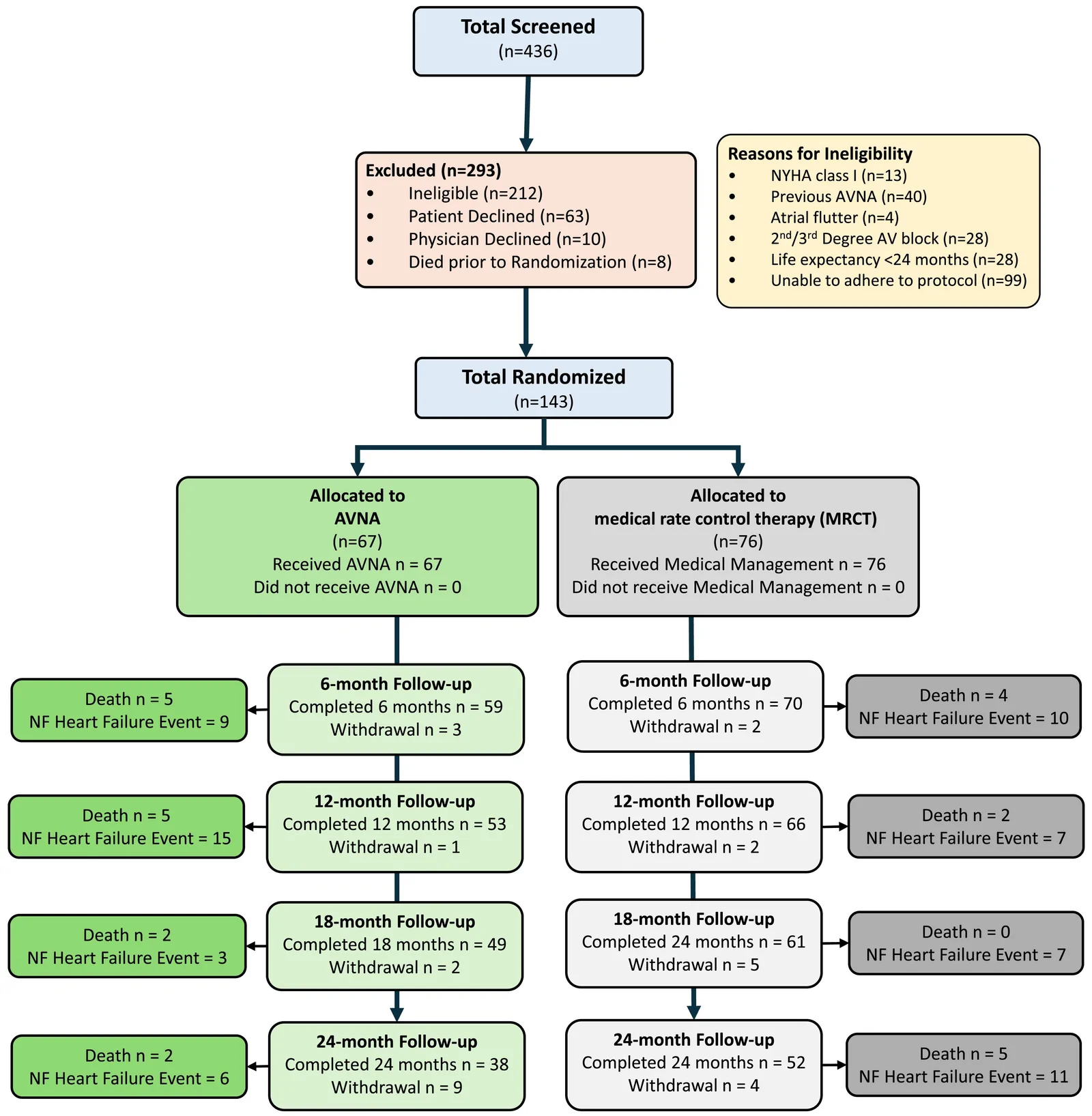
CONSORT

The final study cohort therefore included 143 patients randomized to receive AVNA or MRCT. Reasons for exclusion from randomization are provided in *Figure 1*. After randomization, 67 patients successfully received AVNA whilst 76 received MRCT. There was no crossover between groups. The demographics, clinical characteristics, and baseline medications were well-balanced at the time of randomization (*Table 1*).

**Table 1:** Baseline characteristics

	MRCT (*n* = 76)	AVNA (*n* = 67)
Age (years)	72 ± 7	71 ± 10
Male sex	69 (91)	61 (91)
Body mass index (kg/m^2^)	30 [27–34]	28 [26–33]
Hyperlipidaemia	51 (67)	43 (64)
Diabetes	26 (34)	25 (37)
Hypertension	57 (75)	39 (58)
Obstructive sleep apnoea	20 (26)	17 (25)
Ischaemic cardiomyopathy	37 (49)	32 (48)
Non-ischaemic cardiomyopathy	39 (51)	35 (52)
NYHA class		
II	48 (63)	35 (52)
III	27 (36)	32 (48)
IV	1 (1)	0 (0)
Intrinsic QRS duration (ms)	161 ± 21	158 ± 25
Biventricular paced QRS duration (ms)	152 ± 47	146 ± 50
True LBBB QRS morphology	75 (98.7)	63 (94.0)
Haemoglobin (g/L)	142 ± 16	139 ± 16
Creatinine (µmol/L)	117 ± 32	125 ± 41
eGFR (mL/min/1.73 m^2^)	57 ± 17	53 ± 19
Resting heart rate (bpm)	72 [69–82]	72 [66–80]
Resting systolic blood pressure (mmHg)	119 ± 18	120 ± 16
Resting diastolic blood pressure (mmHg)	74 ± 12	75 ± 12
Biventricular pacing percentage on device (%)	75 [48–91]	89 [69–94]
Biventricular pacing percentage on Holter (%)	74 [51–90]	74 [57–93]
Left ventricular ejection fraction (%)	27 [21–36]	29 [24–36]
Left atrial volume (mL)	115 [91–151]	120 [95–151]
Beta-blockers	72 (95)	66 (99)
ACE-inhibitors/ARB	56 (74)	49 (73)
Mineralocorticoid receptor antagonists	50 (66)	39 (58)
Loop diuretics	65 (86)	57 (85)
Digoxin	27 (37)	32 (48)
Amiodarone	2 (3)	2 (3)
Calcium channel blocker	4 (5)	3 (5)
Pre-existing CRT-D	39 (51)	30 (45)

Data are given as mean ± standard deviation, *n* (%), or median [interquartile range].

ACE, angiotensin-converting enzyme; ARB, angiotensin receptor blocker; CRT-D, cardiac resynchronization therapy with defibrillator; eGFR, estimated glomerular filtration rate; LBBB, left bundle branch block; NYHA, New York Heart Association.

### Primary endpoint

**Figure 2:**
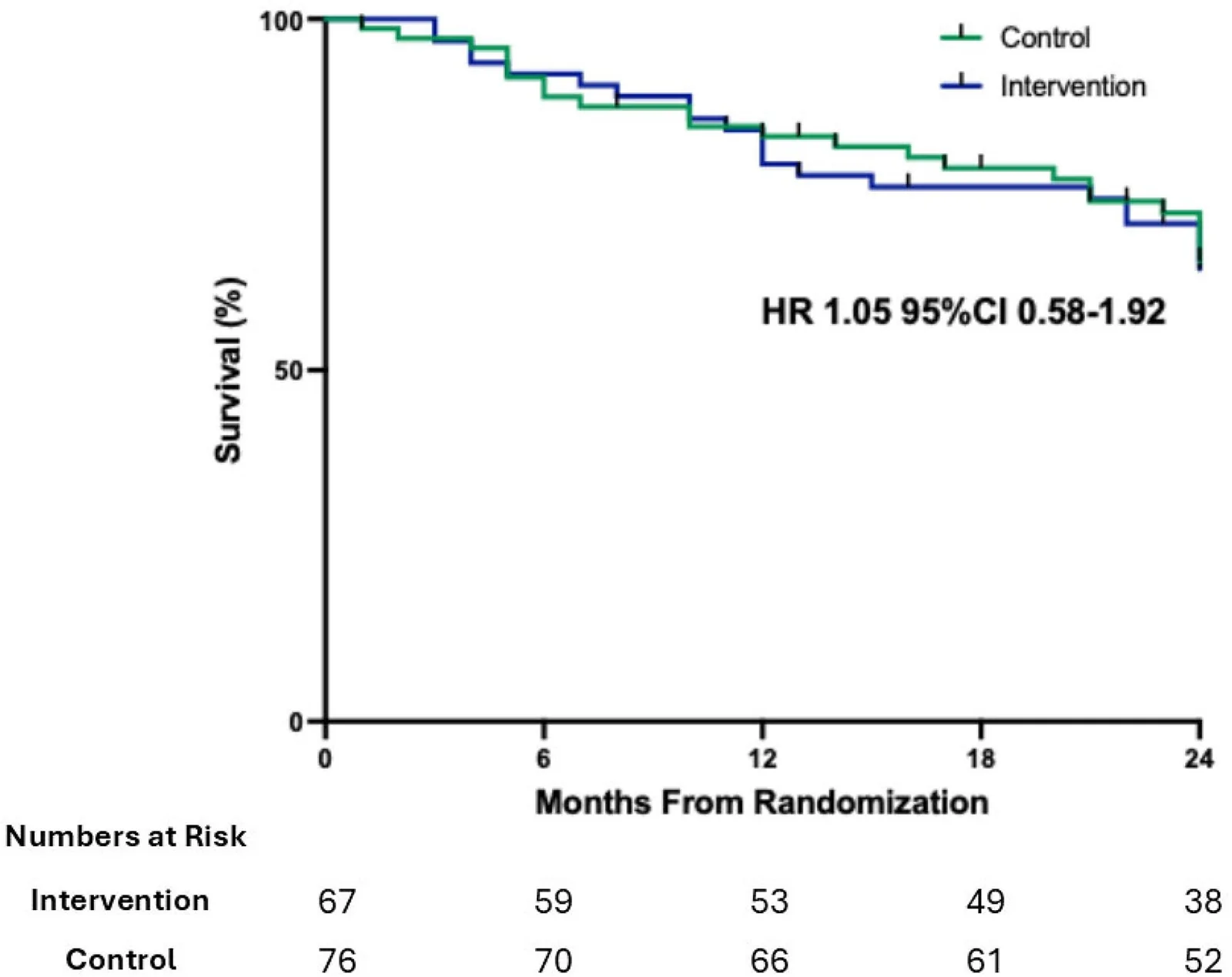
Time-to-first event primary endpoint survival analysis

*Table 2* shows the results of the primary analysis. In total, there were 47 primary composite endpoint events of all-cause mortality or nonfatal HF events in the AVNA group and 46 in the MRCT group (IRR 1.16, 95% CI 0.60–2.24, *P* = .66). There was no difference in the time-to-first event Kaplan–Meier survival analysis (*Figure 2*).

**Table 2:** Primary and secondary outcomes

Outcome	Events		Events per 100-patient years		IRR/OR	95% CI	*P*-value
	MRCT	AVNA	MRCT	AVNA			
Composite primary endpoint	46	47	32.8	36.8	1.16	0.60–2.24	.66
All-cause mortality	11	14	7.8	11	1.56	0.66–3.72	.31
Nonfatal heart failure events	35	33	24.9	25.8	1.07	0.49–2.34	.87
Cardiovascular mortality	5	8	3.6	6.3	1.93	0.60–6.20	.27
Unplanned hospitalizations	104	101	74.1	78.2	1.01	0.71–1.74	.68
Ventricular arrhythmias requiring device therapies	10	6	7.8	4.7	0.68	0.17–2.63	.58

CI, confidence interval; IRR, incidence rate ratio; OR, odds ratio.

### Mortality, hospitalizations, and device therapy

*Table 2* shows the results of secondary mortality and hospitalization endpoints. In total, 14 patients (21%) in the AVNA group and 11 patients (15%) in the MRCT group died (OR 1.56, 95% CI 0.66–3.72), while death attributed to a cardiovascular cause occurred in eight patients (12%) in the AVNA group and five patients (7%) in the MRCT group (OR 1.93, 95% CI 0.60–6.20). Nonfatal HF events occurred on 33 occasions in the AVNA group and 35 occasions in the MRCT group (IRR 1.07, 95% CI 0.49–2.34). Unplanned hospitalizations occurred on 101 occasions in the AVNA group and 104 occasions in the MRCT group (IRR 1.01, 95% CI 0.71–1.74). Device therapy for ventricular arrhythmias was required on 16 occasions in total: AVNA group (*n* = 6) vs. MRCT group (*n* = 10) (IRR 0.68, 95% CI 0.17–2.63). Inappropriate shocks from rapid AF occurred seven times in the MRCT group and none in the AVNA group.

### Biventricular pacing percentage

At final follow-up, total biventricular pacing percentage on device interrogation was higher in the AVNA group compared with the MRCT group [98.0% (89.6–99.0%) vs. 87.4% (72.2–94.2%), *P* = .0006].

### Quality of life, heart failure symptoms, and 6-min walk distances

*Table 3* shows the results of the SF-36 Quality-of-Life Questionnaire. There were no differences in physical health or mental health score groups between the two groups from baseline to 24-month follow-up. Supplementary data online, *Table S6*, MLHF scores, and *Table S7* show the results of the 6MWD at baseline and at 24-month follow-up both not statistically different between groups.

**Table 3:** SF-36 quality-of-life questionnaire

	MRCT		AVNA		*P*-value
	Mean ± SD	95% CI	Mean ± SD	95% CI	
Physical health					
Baseline	51.77 ± 13.44	48.57–54.98	52.68 ± 12.99	49.41–55.95	.33
6 months	54.09 ± 16.24	50.13–58.05	55.80 ± 13.58	52.29–59.31	
12 months	52.09 ± 14.84	48.41–55.77	53.26 ± 13.89	49.58–56.95	
18 months	52.90 ± 14.62	49.09–56.71	54.10 ± 14.74	49.91–58.29	
24 months	53.98 ± 15.21	49.79–58.17	54.27 ± 14.29	49.97–58.56	
Mental health					
Baseline	39.01 ± 5.66	37.67–40.36	37.92 ± 5.59	36.51–39.33	.78
6 months	38.51 ± 6.85	36.84–40.18	37.90 ± 4.73	36.68–39.12	
12 months	38.00 ± 7.19	36.22–39.78	39.54 ± 6.52	37.81–41.28	
18 months	39.12 ± 5.74	37.62–40.62	39.34 ± 5.65	37.73–40.95	
24 months	37.72 ± 6.17	36.02–39.42	38.42 ± 5.79	36.68–40.16	

CI, confidence interval; SD, standard deviation.

### Procedure-related adverse events

There were two procedure-related events reported during the CRT-D implantation: one pocket haematoma requiring revision and one pocket infection requiring hospitalization for intravenous antibacterial therapy and device extraction. There were no reported adverse events related to AVNA.

## Discussion

### Main findings

This multicentre randomized controlled clinical trial investigated the prognostic impact of AVNA vs. MRCT in patients with permanent AF and CRT-D devices. The study was terminated early due to futility. The study found that there was no evidence to support any prognostic benefit of AVNA compared with MRCT despite achieving significantly greater biventricular pacing percentages. We observed no differences in the incidence rate of the primary composite endpoint of all-cause mortality and nonfatal HF events or in key secondary endpoints including cardiovascular mortality, unplanned hospitalizations, ventricular arrhythmias requiring device therapies, quality of life, HF symptoms, or exercise capacity (*Structured Graphical Abstract*). The only difference identified between the two groups was a significantly elevated risk of inappropriate device therapy in the MRCT group. This study provides the first randomized data for AVNA in patients with permanent AF who otherwise meet criteria for CRT-D and refutes previous observational data suggesting a clear prognostic benefit for AVNA in this context.^16^ However, the trial mainly enrolled patients with reasonable rate control and biventricular pacing percentage at baseline, limiting the ability to extend the findings of this trial to CRT-D patients with uncontrolled AF or low biventricular pacing percentage.

### Cardiac resynchronization therapy and atrial fibrillation

Randomized evidence for the use of CRT in patients with AF is lacking as most of the major CRT trials excluded patients with permanent AF. However, substantial data suggest that the presence of AF attenuates the benefits of CRT. A sub-study of the Resynchronization/Defibrillation for Ambulatory Heart Failure Trial (RAFT) showed that patients with the usual criteria for CRT but coexistent permanent AF did not obtain mortality or HF hospitalization benefits from CRT as compared with ICD alone.^13^ A meta-analysis of observational data including 83 571 patients further showed that the presence of AF in patients with CRT was associated with increased all-cause and cardiovascular mortality compared with patients with sinus rhythm, whilst CRT in patients with AF did not decrease mortality when compared with ICD or MRCT alone.^13^

Several potential factors may underscore the lack of CRT-mediated benefit in patients with AF. Firstly, AF may reflect a more advanced cardiac disease. From the observational studies showing lack of CRT benefit in AF compared with sinus rhythm, AF patients often demonstrated older age, broader QRS duration, and more severe HF symptoms.^13^ Secondly, AF is associated with several haemodynamic alterations which may limit the benefit from CRT.^22^ Furthermore, AF also results in a loss of atrioventricular synchrony, an important mediator of the benefit of CRT.^23,24^ Finally, AF is often associated with rapid and irregular activation of the ventricles resulting in the reduction of effective biventricular pacing in patients with a CRT device.

### Atrioventricular node ablation for permanent atrial fibrillation in cardiac resynchronization therapy

Whilst the evidence suggests that AF may attenuate the efficacy of CRT, observational data have suggested that AVNA for AF in this population may improve outcomes. A meta-analysis of the observational data showed a 58% reduction in all-cause mortality associated with AVNA.^16^ However, this first randomized controlled trial investigating AVNA in this population found no evidence that AVNA was able to reduce mortality or nonfatal HF events, despite achieving increased biventricular pacing percentage compared with MRCT.

There are several potential reasons for the findings of this study. First, whilst increasing biventricular pacing percentage has been shown to be critical to the success of CRT in patients with both sinus rhythm and AF, AF patients with optimized biventricular pacing percentages of greater than 98.5% demonstrate poorer outcomes compared with patients with sinus rhythm and suboptimal biventricular pacing percentages of less than 98.5%.^25^ This suggests additional factors beyond biventricular pacing percentages likely contribute to the poor prognosis of patients with AF. Second, patients in both arms were relatively well rate-controlled at baseline, with resting heart rates at 72 bpm and Holter-reported biventricular pacing percentages greater than 70%. While patients with relatively well-controlled heart rate at baseline do not derive prognostic benefit from AVNA, patients with uncontrolled ventricular rates at baseline potentially represent a subgroup of patients who may benefit.

Finally, it should be noted that the overall event rate seen in this trial was 34.9 events per 100 patient-years (32.8 events per 100 patient-years in control and 36.8 events per 100 patient-years in intervention) which is less than the 50 events per 100 patient-years that was expected. This is likely related to the fact that this cohort represented a less symptomatic cohort of patients with mainly NYHA Class II symptoms, rather than NYHA Class III patients as seen in the COMPANION trial, on which this study was powered. In addition, this cohort was well-treated for HFrEF with the majority receiving GDMT based on existing guidelines at trial initiation. These medications are all associated with significant prognostic benefit in patients with HFrEF.^26–29^ Even though more recent additions to HFrEF GDMT including neprilysin inhibitors and sodium–glucose cotransporter 2 inhibitors were not prespecified in this cohort, the use of ACEi, ARBs, beta-blockers, and MRAs would undoubtedly have reduced the event rate across both groups. In this context, AVNA to optimize rate control likely plays less of a role than it may have done previously, and this may explain the different outcome seen in this study compared with the previously published observational studies.^16^

### Clinical implications

CRT-eligible patients with permanent AF currently have a Class IIa indication for an AVNA procedure on the basis of several observational studies showing prognostic benefits.^15^ However, data from this first randomized trial showed no evidence that AVNA confers a prognostic or symptomatic benefit in all CRT-D patients with permanent AF and suggest that clinicians should carefully consider whether this is appropriate in patients with similar baseline characteristics.

### Limitations

This was a relatively small randomized controlled trial which recruited 143 patients over a period of 7 years and was terminated early resulting in imprecise estimates with wide 95% CIs. Whilst the trial found no evidence of benefit for AVNA over MRCT in a relatively unselected cohort of CRT-D patients with permanent AF, the trial does not exclude benefits in certain subcohorts of patients, such as those with rapid ventricular rates at baseline. The vast majority of patients recruited to this trial were male, meaning the findings are unlikely to be generalizable to female populations. Formal interim analyses for efficacy were prespecified in the protocol; at the first interim analysis, the DSMB reviewed blinded event rates across both arms and requested an additional conditional power analysis to assess futility. As a result of the futility analysis, the DSMB recommended early termination of the trial to which the Steering Committee agreed. Although the study did not reach the anticipated sample size, the recruited number was adequate to determine that the trial was unlikely to show a significant difference in primary endpoint between groups. Additionally, enrolling patients through two pathways—those with AF at CRT implant and those who developed AF later—may have introduced heterogeneity and potential bias into the study population, but this was done deliberately as clinical equipoise exists in both clinical contexts.

Finally, this study started recruiting in 2013 at a time when GDMT for HFrEF did not include neprilysin inhibitors or sodium–glucose cotransporter 2 inhibitors. Contemporary management of HFrEF with the addition of these drugs may have had a beneficial impact on the outcomes of this study.

## Conclusions

The CAAN-AF trial compared AVNA to MRCT in CRT-D patients with permanent AF. The trial was stopped early after the first interim analysis indicated a low probability of demonstrating a difference between groups if continued. In this cohort of patients with relatively well-controlled ventricular rates at baseline, AVNA was not associated with a significant reduction in a composite endpoint of all-cause mortality and nonfatal HF events. Additionally, there was no significant reduction in the incidence of cardiovascular mortality, unplanned hospitalizations, or ventricular arrhythmias. This hypothesis-generating trial highlights the need to better identify CRT candidates most likely to benefit from AVNA above MRCT strategies alone.

## Supplementary Material

ehag206_Supplementary_Data

## Declarations

### Data Availability

Collaborative data requests for de-identified participant data underlying the findings of this study will be available from the Centre for Heart Rhythm Disorders, Adelaide University, on reasonable request. Requests should specify the required data fields and the purpose of the request and include a detailed research plan. The statistical analysis plan is provided in appendix, and the data dictionary is available on request. All requests will be considered on a case-by-case basis. Requestors will be required to sign a data sharing agreement with Adelaide University and obtain approval from the institutional Human Research Ethics Committee before data transfer.

### Pre-registered Clinical Trial Number

The pre-registered clinical trial number is NCT0152289.

## References

[1] Chung MK, Patton KK, Lau C-P, Dal Forno ARJ, Al-Khatib SM, Arora V, et al 2023 HRS/APHRS/LAHRS guideline on cardiac physiologic pacing for the avoidance and mitigation of heart failure. Heart Rhythm 2023;20:e17–91. 10.1016/j.hrthm.2023.03.1538 PMC11062890 37283271

[2] Bristow MR, Saxon LA, Boehmer J, Krueger S, Kass DA, De Marco T, et al Cardiac-resynchronization therapy with or without an implantable defibrillator in advanced chronic heart failure. N Engl J Med 2004;350:2140–50. 10.1056/NEJMoa032423 15152059

[3] Cleland JG, Daubert JC, Erdmann E, Freemantle N, Gras D, Kappenberger L, et al The effect of cardiac resynchronization on morbidity and mortality in heart failure. N Engl J Med 2005;352:1539–49. 10.1056/NEJMoa050496 15753115

[4] Moss AJ, Hall WJ, Cannom DS, Klein H, Brown MW, Daubert JP, et al Cardiac-resynchronization therapy for the prevention of heart-failure events. N Engl J Med 2009;361:1329–38. 10.1056/NEJMoa0906431 19723701

[5] Sapp JL, Sivakumaran S, Redpath CJ, Khan H, Parkash R, Exner DV, et al Long-term outcomes of resynchronization-defibrillation for heart failure. N Engl J Med 2024;390:212–20. 10.1056/NEJMoa2304542 38231622

[6] Tang AS, Wells GA, Talajic M, Arnold MO, Sheldon R, Connolly S, et al Cardiac-resynchronization therapy for mild-to-moderate heart failure. N Engl J Med 2010;363:2385–95. 10.1056/NEJMoa1009540 21073365

[7] Gold MR, Daubert C, Abraham WT, Ghio S, St John Sutton M, Hudnall JH, et al The effect of reverse remodeling on long-term survival in mildly symptomatic patients with heart failure receiving cardiac resynchronization therapy: results of the REVERSE study. Heart Rhythm 2015;12:524–30. 10.1016/j.hrthm.2014.11.014 PMC4390984 25460860

[8] Dickstein K, Normand C, Auricchio A, Bogale N, Cleland JG, Gitt AK, et al CRT survey II: a European society of cardiology survey of cardiac resynchronisation therapy in 11 088 patients-who is doing what to whom and how? Eur J Heart Fail 2018;20:1039–51. 10.1002/ejhf.1142 29457358

[9] Wilton SB, Leung AA, Ghali WA, Faris P, Exner DV. Outcomes of cardiac resynchronization therapy in patients with versus those without atrial fibrillation: a systematic review and meta-analysis. Heart Rhythm 2011;8:1088–94. 10.1016/j.hrthm.2011.02.014 21338711

[10] Mustafa U, Atkins J, Mina G, Dawson D, Vanchiere C, Duddyala N, et al Outcomes of cardiac resynchronisation therapy in patients with heart failure with atrial fibrillation: a systematic review and meta-analysis of observational studies. Open Heart 2019;6:e000937. 10.1136/openhrt-2018-000937 PMC6546263 31217991

[11] Buck S, Rienstra M, Maass AH, Nieuwland W, Van Veldhuisen DJ, Van Gelder IC. Cardiac resynchronization therapy in patients with heart failure and atrial fibrillation: importance of new-onset atrial fibrillation and total atrial conduction time. Europace 2008;10:558–65. 10.1093/europace/eun064 18356205

[12] Upadhyay GA, Choudhry NK, Auricchio A, Ruskin J, Singh JP. Cardiac resynchronization in patients with atrial fibrillation: a meta-analysis of prospective cohort studies. J Am Coll Cardiol 2008;52:1239–46. 10.1016/j.jacc.2008.06.043 18926327

[13] Healey JS, Hohnloser SH, Exner DV, Birnie DH, Parkash R, Connolly SJ, et al Cardiac resynchronization therapy in patients with permanent atrial fibrillation: results from the resynchronization for ambulatory heart failure trial (RAFT). Circ Heart Fail 2012;5:566–70. 10.1161/CIRCHEARTFAILURE.112.968867 22896584

[14] Kamath GS, Cotiga D, Koneru JN, Arshad A, Pierce W, Aziz EF, et al The utility of 12-lead Holter monitoring in patients with permanent atrial fibrillation for the identification of nonresponders after cardiac resynchronization therapy. J Am Coll Cardiol 2009;53:1050–5. 10.1016/j.jacc.2008.12.022 19298918

[15] Glikson M, Nielsen JC, Kronborg MB, Michowitz Y, Auricchio A, Barbash IM, et al 2021 ESC Guidelines on cardiac pacing and cardiac resynchronization therapy: Developed by the Task Force on cardiac pacing and cardiac resynchronization therapy of the European Society of Cardiology (ESC) With the special contribution of the European Heart Rhythm Association (EHRA). Eur Heart J 2021;42:3427–520. 10.1093/eurheartj/ehab364

[16] Ganesan AN, Brooks AG, Roberts-Thomson KC, Lau DH, Kalman JM, Sanders P. Role of AV nodal ablation in cardiac resynchronization in patients with coexistent atrial fibrillation and heart failure a systematic review. J Am Coll Cardiol 2012;59:719–26. 10.1016/j.jacc.2011.10.891 22340263

[17] Sanders P, Ariyaratnam JP, Puvrez A, Nicholls SJ, Thomas G, Ganesan A, et al Cardiac resynchronization therapy and AV node ablation in heart failure with reduced ejection fraction and atrial fibrillation. Rationale and design of the CAAN-AF trial. Heart Rhythm 2025;6:1828–36. 10.1016/j.hroo.2025.07.018 PMC12674998 41357267

[18] McMurray JJV, Adamopoulos S, Anker SD, Auricchio A, Bohm M, Dickstein K, et al ESC guidelines for the diagnosis and treatment of acute and chronic heart failure 2012: the task force for the diagnosis and treatment of acute and chronic heart failure 2012 of the European Society of Cardiology. Developed in collaboration with the heart. Eur Heart J 2012;33:1787–847. 10.1093/eurheartj/ehs104 22611136

[19] Cleland JG, Abraham WT, Linde C, Gold MR, Young JB, Claude Daubert J, et al An individual patient meta-analysis of five randomized trials assessing the effects of cardiac resynchronization therapy on morbidity and mortality in patients with symptomatic heart failure. Eur Heart J 2013;34:3547–56. 10.1093/eurheartj/eht290 PMC3855551 23900696

[20] O'Brien PC, Fleming TR. A multiple testing procedure for clinical trials. Biometrics 1979;35:549–56. 10.2307/2530245 497341

[21] Schulz KF, Grimes DA. Multiplicity in randomised trials II: subgroup and interim analyses. Lancet 2005;365:1657–61. 10.1016/S0140-6736(05)66516-6 15885299

[22] Fang F, Lee AP, Yu CM. Left atrial function in heart failure with impaired and preserved ejection fraction. Curr Opin Cardiol 2014;29:430–6. 10.1097/HCO.0000000000000091 25032726

[23] Auricchio A, Stellbrink C, Block M, Sack S, Vogt J, Bakker P, et al Effect of pacing chamber and atrioventricular delay on acute systolic function of paced patients with congestive heart failure. The pacing therapies for congestive heart failure study group. The guidant congestive heart failure research group. Circulation 1999;99:2993–3001. 10.1161/01.CIR.99.23.2993 10368116

[24] Meluzín J, Novák M, Müllerová J, Krejcí J, Hude P, Eisenberger M, et al A fast and simple echocardiographic method of determination of the optimal atrioventricular delay in patients after biventricular stimulation. Pacing Clin Electrophysiol 2004;27:58–64. 10.1111/j.1540-8159.2004.00386.x 14720156

[25] Hayes DL, Boehmer JP, Day JD, Gilliam FR, 3rd, Heidenreich PA, Seth M, et al Cardiac resynchronization therapy and the relationship of percent biventricular pacing to symptoms and survival. Heart Rhythm 2011;8:1469–75. 10.1016/j.hrthm.2011.04.015 21699828

[26] The Cardiac Insufficiency Bisoprolol Study II (CIBIS-II): a randomised trial. Lancet 1999;353:9–13. 10.1016/S0140-6736(98)11181-9 10023943

[27] Pitt B, Zannad F, Remme WJ, Cody R, Castaigne A, Perez A, et al The effect of spironolactone on morbidity and mortality in patients with severe heart failure. Randomized Aldactone Evaluation Study Investigators. N Engl J Med 1999;341:709–17. 10.1056/NEJM199909023411001 10471456

[28] Yusuf S, Pitt B, Davis CE, Hood WB, Cohn JN. Effect of enalapril on survival in patients with reduced left ventricular ejection fractions and congestive heart failure. N Engl J Med 1991;325:293–302. 10.1056/NEJM199108013250501 2057034

[29] Effects of enalapril on mortality in severe congestive heart failure. Results of the cooperative north scandinavian enalapril survival study (CONSENSUS). N Engl J Med 1987;316:1429–35. 10.1056/NEJM198706043162301 2883575

