# Supplementary material for: Cardiac resynchronization therapy with or without atrioventricular node ablation in atrial fibrillation: the CAAN-AF trial
Source: Eur Heart J. 2026 Apr 16;47(36):5128–38. doi: 10.1093/eurheartj/ehag206 (PMC13593208; doi:10.1093/eurheartj/ehag206)
Supplement: Supplementary file 1 [file ehag206_supplementary_data.docx]

**SUPPLEMENTAL MATERIAL**

**Cardiac resynchronization therapy with or without atrioventricular node ablation in atrial fibrillation: the CAAN-AF Trial**

Prashanthan Sanders MBBS, PhD;^1,2^ Jonathan P. Ariyaratnam MB BChir, PhD;^1,2^ Martin K. Stiles MBChB, PhD;^3^ Alexis Puvrez MD, PhD;^1^ Stephen J. Nicholls MBBS, PhD;^4^ Walter P. Abhayaratna MBBS, PhD;^5^ Mark Perrin MBBS;^6^ Jeff Alison, MBBS;^3^ Rajeev K. Pathak, MBBS, PhD;^7^ Anand Ganesan MBBS, PhD;^8^ Vincent Paul, MBBS, PhD;^9^ Stuart P. Thomas, MBBS, PhD;^10^ Gijo Thomas PhD;^1^ Melissa E. Middeldorp MPH, PhD;^1,2^ Elnaz Shahmohamedeh MBBS;^1^ Adrian D. Elliott PhD;^1,2^ Robert Park, MBBS;^11^ Harris Haqqani, MBBS, PhD;^12^ Dean Boddington, MBBS;^13^ Peter M. Kistler, MBBS, PhD;^14^ Paul Martin, MBBCh, PhD;^15^ Raymond Sy, MBBS, PhD;^16^ Stephan Willems, MD;^17^ Paul Stobie, MBBS;^18^ Matthew Webber, MBBS;^19^ Gerald Kaye, MBBS;^20^ Paul MacIntyre, MBBS;^21^ Bobby John, MBBS, PhD;^22^ Daniel Steven, MD;^23^ Azlan Hussin, MD;^24^ Suresh Singarayar, MBBS;^25^ David Whalley, MBBS, PhD;^26^ David O’ Donnell, MBBS;^27^ Nigel Lever, MBBS;^28^ Rajiv Mahajan, MD, PhD;^1,29^ Daniel Ninio, MBBS, PhD;^2^ John Amerena, MBBS, PhD;^6^ Adrian Esterman, PhD;^30^ Derek P. Chew, MBBS, PhD;^4,8^ Matthew Worthley, MBBS, PhD;^1,2^ Andrew Tonkin, MBBS, MD;^31^ Carmine G. De Pasquale, BMBS, PhD;^8^ Jonathan M. Kalman MBBS, PhD^32^

**From:**

^1^Centre for Heart Rhythm Disorders and Discipline of Medicine, University of Adelaide, Adelaide, South Australia, Australia; ^2^Department of Cardiology, Central Adelaide Local Health Network, Adelaide, South Australia, Australia; ^3^ Waikato Clinical School, University of Auckland, Hamilton, New Zealand; ^4^Victorian Heart Institute, Monash University, Melbourne, Victoria, Australia ; ^5^Canberra Hospital and the Australian National University, Canberra, Australian Capital Territory, Australia; ^6^University Hospital, Geelong, Victoria, Australia; ^7^Canberra Heart Rhythm, Canberra, Australian Capital Territory, Australia; ^8^Flinders University and Flinders Medical Centre, Adelaide, South Australia, Australia; ^9^Fiona Stanley Hospital, Perth, Western Australia, Australia; ^10^Westmead Hospital, Sydney, New South Wales, Australia; ^11^Gold Coast Hospital, Gold Coast, Queensland, Australia; ^12^Prince Charles Hospital, Brisbane, Queensland, Australia; ^13^Tauranga Hospital, Tauranga, New Zealand; ^14^Alfred Hospital, Melbourne, Victoria, Australia; ^15^Royal Brisbane Hospital, Brisbane, Queensland, Australia; ^16^Royal Prince Alfred Hospital & Concord Hospital, Sydney, New South Wales, Australia; ^17^Asklepios Clinic St. Georg, Hamburg, Germany; ^18^Sir Charles Gairdner Hospital, Perth, Western Australia, Australia; ^19^Capital & Coast Wellington Hospital, Wellington, New Zealand; ^20^Princess Alexandria Hospital, Brisbane, Queensland, Australia; ^21^Royal Hobart Hospital, Hobart, Tasmania, Australia; ^22^Townsville Hospital, Townsville, Queensland, Australia; ^23^Department of Electrophysiology, University Hospital Cologne, Cologne, Germany; ^24^National Heart Institute, Kuala Lumpur, Malaysia; ^25^Prince of Wales Hospital, Sydney, New South Wales, Australia; ^26^Royal North Shore Hospital, Sydney, New South Wales, Australia; ^27^Austin Health, Melbourne, Victoria, Australia; ^28^Auckland City Hospital, Auckland, New Zealand; ^29^Lyell McEwin Hospital, Adelaide, South Australia, Australia; ^30^University of South Australia, Adelaide, South Australia, Australia; ^31^Monash University, Melbourne, Victoria, Australia; ^32^Melbourne Health and University of Melbourne, Melbourne, Victoria, Australia.

**Contents:**

1. Supplementary Methods
2. Table S1 – Inclusion and Exclusion Criteria
3. Table S2 - Device Lead Positioning
4. Table S3 – Follow-up Assessments
5. Table S4 – Site-specific recruitment
6. Table S5 – Futility Analysis
7. Table S6 – Minnesota Living with Heart Failure Questionnaire
8. Table S7 – Six Minute Walk Distance
9. Figure S1 – Enrolment Pathways (Pathway A and B)

**Supplementary Methods**

**CRT-D Implantation**

Choice of CRT-D system (pulse generator and leads) manufacturer was left to the discretion of the implanting physician. Investigators were instructed to position the left ventricular lead trans-venously via a coronary sinus tributary to pace the lateral or posterolateral wall of the left ventricle, with radiographic documentation of final placement. Positioning of the right ventricular defibrillator lead was determined by the treating physician.

**CRT-D Programming and Optimisation**

Optimisation of atrio-ventricular and inter-ventricular synchrony using 12-lead ECG and cardiac imaging was allowed as clinically determined by the treating physician. Back-up pacing rate was set at 60 beats-per-minute and rate response was switched on for all participants. Ventricular arrhythmia therapy zones were programmed as previously recommended.^19^ The protocol mandated optimisation of CRT-D devices to encourage biventricular pacing in the presence of AF. Specific device-specific programmable algorithms were used to encourage biventricular pacing (e.g. conducted AF response, ventricular sensed response, biventricular trigger, and ventricular rate regulation).

**AV Node Ablation Procedure**

The procedure was performed endocardially using catheter ablation as was routine at each participating centre. In general, this was done on uninterrupted anticoagulation and using ultrasound-guided femoral vein puncture. Radiofrequency catheter ablation of the compact AV node was undertaken guided by intracardiac electrocardiograms and fluoroscopy. Choice of ablation catheter and ablation settings were left to the discretion of individual physician preference. The endpoint of ablation was complete AV block after catheter ablation and a 10-minute waiting period. Cardiac electrograms confirming successful AVNA was confirmed by independent adjudication.

**SF36 Questionnaire**

The SF-36 questionnaire consists of 36 items that assess eight key health domains: physical functioning (e.g., ability to perform daily activities), role limitations due to physical health (e.g., difficulty working), role limitations due to emotional problems (e.g., accomplishing less due to mood), energy/fatigue (e.g., feeling tired or full of energy), emotional well-being (e.g., feeling calm or downhearted), social functioning (e.g., interference with social activities), pain (e.g., intensity and interference), and general health perceptions. Responses are transformed into scores from 0 to 100, with higher scores indicating better health. These domain scores are then grouped to reflect two overall summary measures: Physical Health and Mental Health status, offering a comprehensive view of an individual’s well-being.

**MLWHF Questionnaire**

The Minnesota Living with Heart Failure Questionnaire (MLHFQ) is a validated, disease-specific tool designed to assess the impact of heart failure on a patient’s quality of life. It includes 21 questions covering physical, emotional, and social dimensions affected by heart failure symptoms such as fatigue, breathlessness, and swelling. Patients rate how much each issue has affected their daily life over the past month using a scale from 0 (no effect) to 5 (very much). Scores are totaled to give an overall score ranging from 0 to 105, with higher scores indicating poorer quality of life. The MLHFQ has been widely validated in clinical and research settings, demonstrating strong reliability and sensitivity to changes in a patient’s condition.

**Statistical Analyses**

Continuous variables were reported as mean (standard deviation) for normally distributed data or median (inter-quartile range) for non-normally distributed data. Categorical variables were reported as frequencies and percentages. Continuous variable data was assessed for normality assumptions using the Shapiro-Wilk Test. Continuous data were compared between groups using the student’s t-test or Mann-Whitney U-test as appropriate. Categorical variables were compared between groups using the chi-square test or Fisher’s exact test (when cell size was <5). Time-to-event analyses were undertaken by the Kaplan-Meier survival analysis approach with Hazard ratios and confidence intervals calculated using the Mantel-Haenzsel approach.

**Table S1 – Inclusion and Exclusion Criteria**

**Inclusion Criteria**

- At least 18 years old
- Permanent* AF
- NYHA class II, III or ambulatory class IV HF
- Documented evidence of LVEF ≤35% on echocardiogram or CMR.
- QRS duration on 12 lead ECG ≥120ms
- Able and willing to provide informed consent and comply with all pre, post, and follow up testing, requirements

**Exclusion Criteria**

- < 18 years
- Pregnancy
- Previous AV node ablation
- Second or third-degree AV block
- Inability to provide consent
- Life expectancy less than 24 months due to co-morbid illness other than heart failure e.g. cancer, end stage renal disease or liver failure
- Paroxysmal AF that self-terminates within 7 days

**Table S2 – Device Lead Positioning**

|  | **Control**  **(n=76)** | **Intervention**  **(n=67)** |
| --- | --- | --- |
| **Left ventricular lead** |  |  |
| **Anterior** | 0 (0) | 1 (2) |
| **Antero-Lateral** | 8 (11) | 5 (8) |
| **Lateral** | 27 (36) | 26 (39) |
| **Postero-Lateral** | 26 (35) | 24 (36) |
| **Posterior** | 3 (4) | 1 (2) |
| **Other** | 6 (8) | 5 (8) |
| **Not available** | 5 (7) | 5 (8) |
| **Right ventricular lead** |  |  |
| **Apex** | 51 (68) | 33 (49) |
| **Septal** | 17 (23) | 23 (34) |
| **RVOT** | 3 (4) | 4 (6) |
| **Other** | 3 (4) | 3 (5) |
| **Not available** | 1 (1) | 4 (6) |

Data is n (%)

RVOT = right ventricular outflow tract

**Table S3 – Follow-Up Assessments**

| **Assessment** | **Follow-up** | | | |
| --- | --- | --- | --- | --- |
|  | **6 month** | **12 month** | **18 month** | **24 month** |
| Current Drug Therapy | ✔︎ | ✔︎ | ✔︎ | ✔︎ |
| Physical Examination | ✔︎ | ✔︎ | ✔︎ | ✔︎ |
| Adverse Events post consent | ✔︎ | ✔︎ | ✔︎ | ✔︎ |
| Device Interrogation | ✔︎ | ✔︎ | ✔︎ | ✔︎ |
| 6MWT | ✔︎ | ✔︎ | ✔︎ | ✔︎ |
| NT-proBNP | ✔︎ | ✔︎ |  | ✔︎ |
| Blood Samples (Hb, INR, Sodium, Creatinine, Potassium, eGFR) | ✔︎ | ✔︎ | ✔︎ | ✔︎ |
| Questionnaires SF- 36, CES-Depression Scale, MLHF | ✔︎ | ✔︎ | ✔︎ | ✔︎ |
| Echocardiogram (TTE) | ✔︎ | ✔︎ |  | ✔︎ |
| 24-Hour Holter | ✔︎ |  |  |  |

**Table S4 – Recruitment Numbers at Each Site**

| **Centre** | **City** | **Country** | **Number Recruited** |
| --- | --- | --- | --- |
| **Royal Adelaide Hospital** | Adelaide | Australia | 28 |
| **Flinders Medical Centre** | Adelaide | Australia | 3 |
| **Royal Melbourne Hospital** | Melbourne | Australia | 3 |
| **Monash Medical Centre** | Melbourne | Australia | 12 |
| **Alfred Hospital** | Melbourne | Australia | 5 |
| **Austin Health** | Melbourne | Australia | 1 |
| **University Hospital Geelong** | Melbourne | Australia | 15 |
| **Royal Prince Alfred Hospital** | Sydney | Australia | 2 |
| **Concord Hospital** | Sydney | Australia | 2 |
| **Prince of Wales Hospital** | Sydney | Australia | 1 |
| **Royal North Shore Hospital** | Sydney | Australia | 1 |
| **Royal Perth Hospital** | Perth | Australia | 2 |
| **Fiona Stanley Hospital** | Perth | Australia | 2 |
| **Sir Charles Gairdner Hospital** | Perth | Australia | 3 |
| **Prince Charles Hospital** | Brisbane | Australia | 6 |
| **Princess Alexandria Hospital** | Brisbane | Australia | 2 |
| **Royal Brisbane Hospital** | Brisbane | Australia | 4 |
| **Canberra Hospital** | Canberra | Australia | 9 |
| **Royal Hobart Hospital** | Hobart | Australia | 2 |
| **Townsville Hospital** | Townsville | Australia | 1 |
| **Gold Coast Hospital** | Gold Coast | Australia | 7 |
| **Tauranga Hospital** | Tauranga | New Zealand | 6 |
| **Waikato Hospital** | Waikato | New Zealand | 18 |
| **Capital & Coast Wellington Hospital** | Wellington | New Zealand | 2 |
| **Auckland City Hospital** | Auckland | New Zealand | 1 |
| **Asklepios Clinic St. George** | Hamburg | Germany | 3 |
| **University Hospital Cologne** | Cologne | Germany | 1 |
| **National Heart Institute** | Kuala Lumpur | Malaysia | 1 |

**Table S5 – Futility Analysis**

|  | **Based on current data** | | | | **Sample size: re-estimated** | |  |
| --- | --- | --- | --- | --- | --- | --- | --- |
|  | **Proportions** | | **Conditional power** | **Futility** | **Expected power** | **n/group** | **Futility** |
|  | **Control** | **Intervention** |  |  |  |  |  |
| **Death** | 0.14 | 0.20 | 0.136 | 0.863 | 0.51 | 295 | 0.863 |
| **HF** | 0.26 | 0.22 | 0.059 | 0.940 | 0.36 | 295 | 0.941 |
| **Death/HF** | 0.31 | 0.32 | 0.027 | 0.973 | 0.28 | 314 | 0.972 |

**Table S6 – Minnesota Living with Heart Failure Questionnaire**

| **Control** | | | | **Intervention** | | | | **P Value*** |
| --- | --- | --- | --- | --- | --- | --- | --- | --- |
|  | **mean** | **SD** | **95%CI** |  | **mean** | **SD** | **95%CI** |  |
| **Baseline** | 37.71 | 21.54 | 32.49-42.92 | **Baseline** | 37.57 | 23.13 | 31.59-43.54 | 0.987 |
| **6 Months** | 34.74 | 24.06 | 28.78-40.70 | **6 Months** | 32.07 | 20.88 | 26.67-37.46 |  |
| **12 Months** | 33.90 | 22.69 | 28.19-39.62 | **12 Months** | 30.18 | 22.46 | 24.22-36.13 |  |
| **18 Months** | 34.68 | 23.88 | 28.35-41.02 | **18 Months** | 33.80 | 22.78 | 27.33-40.27 |  |
| **24 Months** | 33.55 | 23.28 | 27.00-40.10 | **24 Months** | 36.61 | 22.27 | 30.00-43.22 |  |

**Table S7 – Six Minute Walk Distance**

| **Control** | | | | **Intervention** | | | **P Value*** |
| --- | --- | --- | --- | --- | --- | --- | --- |
|  | **mean** | **SD** | **95% CI** | **mean** | **SD** | **95% CI** |  |
| **Baseline** | 364.85 | 326.45 | 289.22-440.48 | 337.70 | 118.85 | 307.77-367.63 | 0.186 |
| **24 Months** | 322.76 | 114.26 | 288.43-357.08 | 364.71 | 130.78 | 321.72-407.70 |  |

**Figure S1**
